# Increasing incidence of peanut allergy doubled in children from Bosnia and Herzegovnia during last decade

**DOI:** 10.1186/2045-7022-1-S1-P29

**Published:** 2011-08-12

**Authors:** Adnan Bajraktarevic, Milan Mikovic, Andrea Pahor Kurilic, Semira Penava, Begler Begovic, Amina Selmovic, Zlatko Guzin, Teodora Frankic, Jasna Gutic, Aida Djulepa Djurdjevic, Zeljko Roncevic, Lutvo Sporisevic, Besima Rakic Prnjavorac

**Affiliations:** 1 Public Health Institution of Canton Sarajevo, Pediatrics Department, Sarajevo, Bosnia and Herzegovina; 2 Clinical Medical Center Sarajevo, Clinical Pharmacology, Sarajevo, Bosnia and Herzegovina; 3 Pediatrics Clinic Sarajevo, Department for allergology and pulmonology, Sarajevo, Bosnia and Herzegovina; 4 City Hospital Mostar, Emergency Department, Mostar, Bosnia and Herzegovina; 5 Pharmacy Faculty Sarajevo, Department for Clinical Pharmacology, Sarajevo, Bosnia and Herzegovina; 6 General Hospital Sarajevo, Emergency Department, Sarajevo, Bosnia and Herzegovina; 7 Pediatrics Hospital Mostar, Cardiology Department, Mostar, Bosnia and Herzegovina; 8 First Medical Aid New Sarajevo, Pediatrics Department, Sarajevo, Bosnia and Herzegovina; 9 Pediatrics Health Center Tesanj, General Pediatrics Department, Tesanj, Bosnia and Herzegovina

## Background

Peanut butter is known as a healthy food . It is a type one hypersensitivity reaction to dietary substances from peanuts causing an overreaction of the immune system which in a small percentage of children may lead to severe physical symptoms. Peanut allergy is one of the most common causes of anaphylaxis, a pediatrics medical emergency that requires treatment with an epinephrine (adrenaline).

## Aims

Objective of this study to determine the current frequency of accidental exposures occurring in peanut-allergic children and identify factors associated with exposure.

## Methods

Popular confections include salted peanuts, peanut butter (sandwiches, candy bars, and cups), peanut brittle, and shelled nuts (plain/roasted). Medical charts of children evaluated and diagnosed as having peanut allergy in Pediatrics primary, secondary, tertiary care and allergy and immunology clinic were reviewed during first decade of twenty first century in Bosnia and Herzegovina.

## Results

Peanut allergy is a major cause of food-induced anaphylaxis, with increasing prevalence in Bosnia and Herzegovina as worldwide. Egg allergy is very common in peanut-allergic patients, and sesame seeds should perhaps be considered one of the major food allergens. Our study showed that from 2000 to 2010, the incidence of peanut allergy doubled in children from Bosnia and Herzegovina.

## Discussion

Children avoiding peanut were more likely to have recurrence of their peanut allergy than those ingesting peanut on a regular basis. The exact cause of someone developing a peanut allergy is unknown.

## Conclusions

Peanut allergy is common, potentially severe and rarely resolves causing impaired quality of life in children. Conventional subcutaneous-injection allergen immunotherapy using crude peanut extract is not a recommended treatment because of the risk of severe side effects, largely as a result of specific IgE antibodies. Avoidance and correct identification of the nuts to which a child is allergic should be part of an overall educational plan.

## Key Words

Allergy, Children, Peanut, Incidence, Prevalence.

